# (Re)Conceptualizing decision-making tools in a risk governance framework for emerging technologies—the case of nanomaterials

**DOI:** 10.1007/s10669-022-09870-2

**Published:** 2022-07-24

**Authors:** Martin Mullins, Martin Himly, Isabel Rodríguez Llopis, Irini Furxhi, Sabine Hofer, Norbert Hofstätter, Peter Wick, Daina Romeo, Dana Küehnel, Kirsi Siivola, Julia Catalán, Kerstin Hund-Rinke, Ioannis Xiarchos, Shona Linehan, Daan Schuurbiers, Amaia García Bilbao, Leire Barruetabeña, Damjana Drobne

**Affiliations:** 1 Transgero Limited, Cullinagh, Newcastle West, Co., Limerick, Ireland; 2 Department of Accounting and Finance, Kemmy Business School, University of Limerick, Limerick, Ireland; 3 Department of Biosciences, Paris Lodron University of Salzburg (PLUS), 5020 Salzburg, Austria; 4 GAIKER Technology Centre, Basque Research and Technology Alliance, (BRTA) ES, Gipuzkoa, Spain; 5 Particles-Biology Interactions Laboratory, Empa, Swiss Federal Laboratories for Materials Science and Technology, Lerchenfeldstrasse 5, 9014 St. Gallen, Switzerland; 6 Department Bioanalytical Ecotoxicology (BIOTOX), Helmholtz Centre for Environmental Research - UFZ, Permoserstraße 15, 04318 Leipzig, Germany; 7 Finnish Institute of Occupational Health, Työterveyslaitos, Box 40, 00032 Helsinki, Finland; 8 Department of Anatomy, Embryology and Genetics, University of Zaragoza, Saragossa, Spain; 9 Fraunhofer Institute for Molecular Biology and Applied Ecology IME, Auf dem Aberg 1, 57392 Schmallenberg, Germany; 10 Research Lab of Advanced Composite, Nanomaterials, and Nanotechnology (R-NanoLab), School of Chemical Engineering, National Technical University of Athens, 9 Heroon Polytechniou str, 15780 Zographos, Athens Greece; 11 Management, Cairnes School of Business and Economics, National University of Ireland Galway, Galway, Ireland; 12 De Proeffabriek Josef Israelslaan 63, NL-6813 JB Arnhem, The Netherlands; 13 Department Biology, Biotechnical Faculty, University of Ljubljana, Ljubljana, Slovenia

**Keywords:** Risk governance, Decision-making tools, Risk assessment, Risk management, Nanotechnology, Data quality

## Abstract

The utility of decision-making tools for the risk governance of nanotechnology is at the core of this paper. Those working in nanotechnology risk management have been prolific in creating such tools, many derived from European FP7 and H2020-funded projects. What is less clear is how such tools might assist the overarching ambition of creating a fair system of risk governance. In this paper, we reflect upon the role that tools might and should play in any system of risk governance. With many tools designed for the risk governance of this emerging technology falling into disuse, this paper provides an overview of extant tools and addresses their potential shortcomings. We also posit the need for a data readiness tool. With the EUs NMP13 family of research consortia about to report to the Commission on ways forward in terms of risk governance of this domain, this is a timely intervention on an important element of any risk governance system.

## Introduction

The risk governance (RG) of nanomaterials (NM) use has been at the vanguard of wider governance challenges around emerging technologies. In many respects it represents a *classic* case in that we have a technology with immense economic value and a scientific field replete with scientific uncertainty. One of the main objectives of funded research in the field of nanotechnology has been to develop tools to aid stakeholders in their decision making within a wider governance framework. This is a complex challenge in part because there is no *one size fits all* solution given the diversity of nanotechnology activities and the stakeholders involved. Moreover, the pace of nanotechnology development has outstripped the ability of regulators to foster adequate RG in the domain (Trump et al 2020). A central argument of this paper is that more attention is required on the intrinsic characteristics of decision-making tools. Three central issues arise, firstly those using tools may be unaware of the technical limitations and flawed assumptions underpinning such artefact. Keisler and Linkov (2021) have identified such shortcomings in the use of multi-criterial-decision analysis (MCDA)-based tools. Second, there is the issue of how such tools may have a certain set of *values* embedded within them and users may be unaware of any implicit bias. Thirdly, there is the question of who the tools are designed for and the related notion of inclusivity in the overall RG process. Given the redundancy of many of the nanotechnology-related governance tools over the past two decades it is clear that the creation of such tools is not a simple panacea. It is clear that shortcoming in RG can hamper the economic potential of emerging technologies such as NMs (Isigonis et al. 2020). Decision-making tools are now an important component of such a governance system and it is this element of RG that this paper interrogates. Overall, the goal is to assure that RG is based on transparency, effectiveness, efficiency, accountability, strategic focus, sustainability, equity and fairness as well as respect for the rule of law. Clearly, the chosen solution must be politically and legally realizable as well as ethically and publicly acceptable (Renn 2006). With these global ambitions, an important question that arises is how to navigate the selection of tools designed to address RG challenges.

The objective of this paper is to provide a fuller and more nuanced understanding of decision-making tools from a multidisciplinary perspective. Our team of 18 experts all working on the issue of the RG of NMs has scoped out the various uses of such tools and their origins. At the same time, the paper leveraged the interdisciplinarity of the team to posit a more complete understanding of such tools and how they might assist or indeed hinder better decision-making in the field of RG. Making decision around the risks pertaining to emerging technologies is a political act with societal consequences. Hence, we need to reflect upon how decision-making tools might impact on this process. As a consortium responding to the NMBP-13 call in which the European Commission sought improved RG structures around nanotechnology, NANORIGO has sought to focus on the vexed question of the wider socio-ethical issues around nanotechnology. Thus, we examine decision making tools not just in terms of efficacy but also in terms of how such tools can have embedded assumptions and indeed reify power relations in a particular field. In the sociological and management literature, there is an acceptance of such downsides to the use of tools as aids to decision-making. NANORIGO is charged with creating improved governance around nanotechnology, the goal of this paper is to reflect upon the role decision-making tools can have in this enterprise. The contribution of this paper lies with its ability to broaden the discussion around the use of tools in the different tasks that together form governance. The intrinsic complexity of tools in terms of social relation is largely not reflected upon in the nanotechnology risk literature. This paper also addresses this lacuna.

The task of the European Commission NMBP-13-funded family of consortia is to create an architecture and support (re) use of data, information and knowledge from previously funded projects in the area of NM safety for effective RG. We use the term family advisedly here as the three projects have established close relations over the past three years.^1^ A key aspect of the challenge is how to integrate scientific knowledge, stakeholders’ needs and societal engagements into a RG process. The notion of governance includes the involvement of European citizenry and indeed global citizens in decisions around the risk management of NM related activities. Isigonis et al. (2020) argue that governance is largely informed by the joint actions of risk analysis (including measures of risk prevention, mitigation, or transfer) and risk communication and these elements are captured in this paper. Additionally, one of the goals of our work is to empower citizens and non-experts in the complex RG decision making processes and integrate them into the process. This speaks to the main issue of the roles and responsibilities of experts and the public in a modern democracy (Mark 2005). The authors of this paper are members of the NANORIGO consortium, which has a high degree of focus on overarching questions around RG as a practice and on the creation of a council-like entity or architecture to assist the stakeholders in the challenge of RG. The term RG brings in its wake a set of other concepts such as legitimacy, inclusiveness and reflectiveness signalling an awareness of the contours of power relations (Smart 1994; Renn and Klinke 2015). As a normative goal, RG seeks to create a system to support decision-making that creates a higher degree of trust and trustworthiness both within the expert community and within the wider society. Vallor (2016) in her *Technology and the Virtues* points out that in the early twenty-first century there has been a shift in how philosophers engage with the ethics of technology with a turn towards a *less* essentialist view on the relationship between human beings and technology towards more specificity and empirically led approaches. The right kind of tools could be a key enabler of this approach and for a nuanced set of RG processes, and in this instance, tools for the RG of nanotechnology-related activities.

RG, which is a locus for NANORIGO, is about creating an architecture where informed and participatory options for RG could be elaborated and proposed to decision-makers in line with a multiplicity of ethical guidelines. This was a basis for the European Commission’s (2008) ‘*Code of Conduct for Responsible Nanoscience and Nanotechnologies Research*’. The aim of these rules is to provide Member States, employers, researchers and more generally, individuals and civil society organizations involved or interested in nanoscience and nanotechnologies with an approach to research and development. Responsible nanotechnologies is addressed by the “nano-safety” community, which includes those drawn from the scientific community, ethicists, sociologists and business school academics. The interdisciplinarity implicit here allows for a fruitful engagement between applied ethics and *natural /technical science.* This empirical turn is in line with Aristotelian notions of virtue and its relationship to competence (Fraser 1990; Hackett and Wang 2012).

Recently published works in nanotechnology-related journals provide a useful snapshot of the extant of tools supporting knowledge based decision making that are in operation and/or have been created by research scientists over the last decade or so. From the caLIBRAte project, Isigonis et al. (2019) provided a comprehensive capture of the many tools that have been created for risk communication, evaluation and mitigation around NM-related activities. Trump et al. (2018) produced a taxonomy of six tools groups based on two main types of activities: traditional risk-based and second, a tool that utilizes multi-criteria assessment. From the perspective of the author, RG is not comprised solely of the operation and outputs of tools but also the approaches addressing the socio-cultural dimension too. This includes the problem-solving capacities of individual actors, be they government, the scientific community, industry, Non-Governmental Organizations (NGOs) or civil society as a whole. The goal is to assure that RG is based on transparency, effectiveness, efficiency, accountability, strategic focus, sustainability, equity and fairness, respect for the rule of law and the need for the chosen solution to be politically and legally realizable as well as ethically and publicly acceptable (Renn 2006). An important question that arises is how to navigate the selection of tools designed to address RG challenges.

**Fig. 1:**
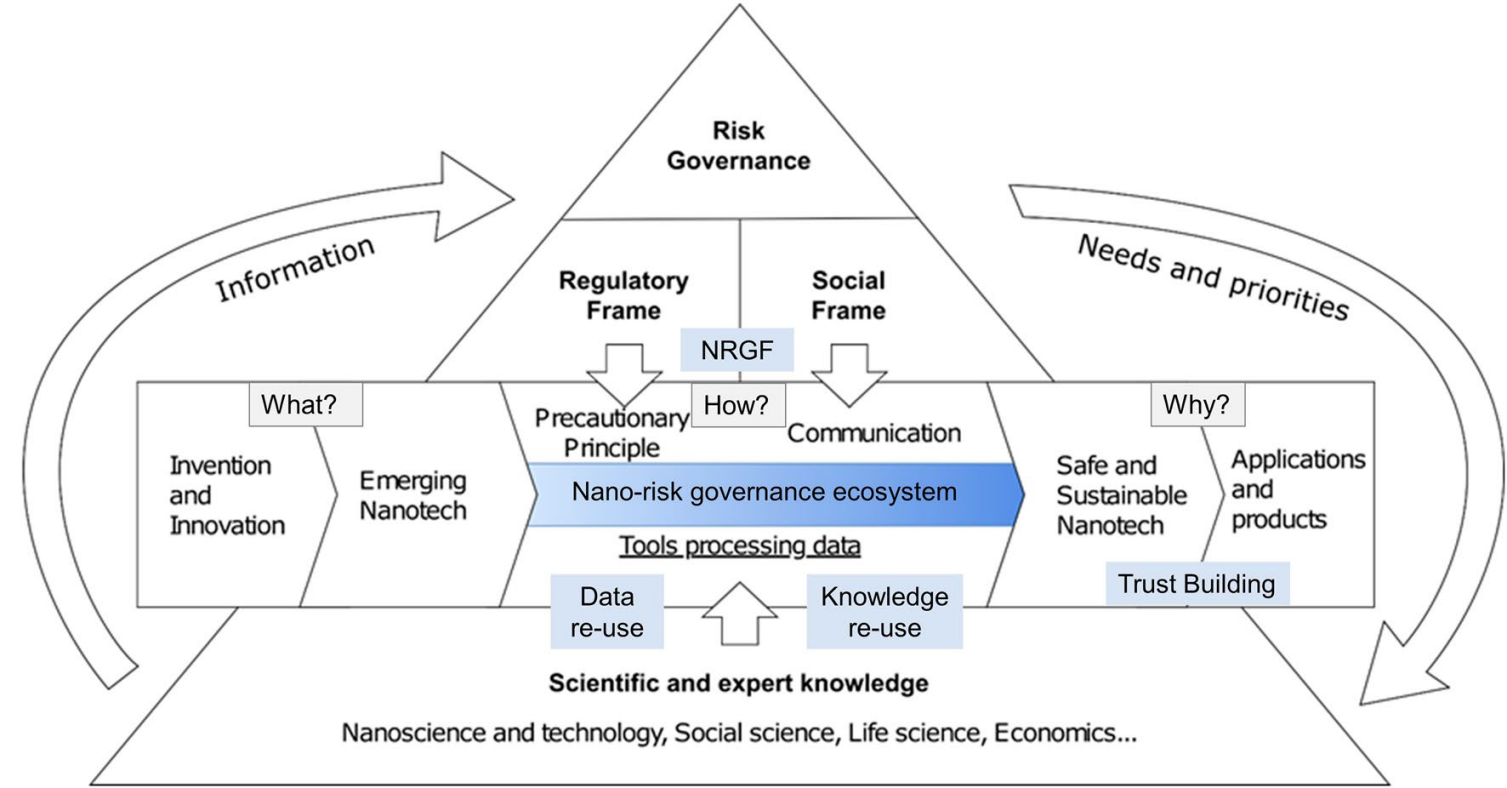
The RG process displaying important elements discussed in the latter part of this perspective article. “Risk Governance” sits at the apex of the triangle functions both a framing concept and an actual set of practices. NRGF denotes nanotechnology risk governance framework and is a key output of the Nanorigo project

What is less than clear in the current literature is how such tools might fit into the overall goals underpinning the ambition to translate theoretical understandings of an RG Framework (RGF) into an analytical framework (Fig. 1). Here, we outline the RG network topography (network configuration) to logically position and distribute a variety of tools addressing nano-risk.

We move forward on the question *“*how to navigate the selection of tools to address RG challenges” by being aware of the presence of multiple “logics” in the field of NM decision-making and governance (Besharov and Smith 2014; Granqvist and Ritvala 2016). The diversity of stakeholders in the community working on nanotechnology governance is striking and includes industry, civil society regulators and academics from a number of disciplines. This characteristic often goes unremarked and does point to a need for a more nuanced understanding of decision making processes. A key set of debates in the decision-making literature revolve around the question of rational thinking and the introduction of more nuanced, less essentialist, ideas of human behaviour in the form of *bounded rationality* (March 1978; Arthur 1994; Dequech 2001). Recent literature in the field has stressed the transactive rationality as a model rationality that, by integrating scientific, democratic, moral and ecological considerations, serves as a more holistic, explanatory and normative guide for public policy and democratic practice (Kuruvilla and Dorstewitz 2010). Here professional backgrounds and organizational functions play an important role in *ways of seeing and thinking* (Berger 1972). From the discipline of political science, there are stronger challenges to the notion that agents embedded in organizations can be expected to make purely rational decisions, especially where the interests of exogenous actors are the focus. An important set of questions emerges here around the purpose of NM decision-making tools and how it relates to a meta-goal of rational decisions in organizational life. If we take on board the Eisenhardt and Zbaracki (1992) position that generally, decision-making is chaotic or stochastic, we might posit the question—is the purpose of the multitude of NM tools to improve this situation. If this is the case, what are the implications for the wider ambitions of RG?

The decision-making literature and adjacent work also provide us with a nuanced view on conceptualizing such tools. According to Thornton and Ocasio (2008) decision makers take into account issues related to the *dominant* logic dismissing those associated with any other logic. We draw on the concept of logics here as there are a number of distinct logics present in the communities working on the RG of nanotechnologies.^2^ The embedding of certain dispositions in an RG tool can imperceptibly perpetuate the dominance of one organising logic. The creation and operation of such tools introduces this socio-materiality into the field and leads us to questions about the impact of the selection of such tools (Waelbers 2009). The debates around decision making have intensified more recently with the advent of Artificial Intelligence (AI) and the use of big data. Within the parameters of this paper and in very large brush strokes, decision-making tools can be created in such a way as to reinforce existing power relations, alternatively we take on board Latour’s position that in late modernity we see morality embedded in services and artefacts (Latour 1992; Latour and Venn 2002). The point here is that NANORIGO’s holistic vision around RG means that we do not want to reinforce such power relations. Based on technological and social changes since the 1960s, social theorists and sociologists describe contemporary societies as a continuation of modernity, a post-modern type of society, second modernity or late modernity (Turner and Turner 1990, Lash 2003, Latour 2003, Castells 2007, Castells 2008, Castells 2014, Lash 2018). Closely related terms are post-industrial society, post-modern society, computer society, information society, knowledge society, telematic society, society of the spectacle (postmodernism), network society or even “*liquid modernity*” (Castells 2014). An interesting juxtaposition here is implicit in Bauman’s Liquid Modernity (Bauman 2000) and that is such tools may inhibit further agency and responsibility of human actors. The creation of such tools is an attempt at engineering rationality and with it comes effects. However, Ulrich Beck and Anthony Giddens describe society as new reflexive modernity with the emergence of the so-called risk society, where reflexive modernization means self-confrontation with the effects of risk society (Beck 1994). If this interpretation of society is taken, the selection of tools should be based on the premise that they allow informed and reflexive decision-making.

For many stakeholders, the field of NM risk is noisy and overwhelming (Joubert et al. 2020; Tengler et al. 2020; Murphy et al 2017). In such cases, the extant tools may assist as effective risk communication tools and hence generate a *virtu* effect. It could also be the case that a certain rationality could be achieved through the “doing” around the tools; that is to say, the operation of tools affords a reflective moment in itself. That said, the decision-making literature cannot be accused of naiveté around how people embedded in large hierarchical organizations make decisions (Jones 1991). Moreover, work on decision-making tools alerts us to the potential for bias and reification in such tools. We need therefore to reflect on how the use of tools influences RG and how to conceptualize or position such tools in any RGF. This paper is the result of reflections on the part of a group of academics from across Europe working on Work Package 2 in Nanorigo which is focused on the use and development of RG tools. The team of authors is drawn from a range of disciplines from chemistry and toxicology to informatics, through to specialists on governance (social sciences). Thus, the paper reflects upon the nature of decision-making tools and their role on the RG of nanotechnology related activities. Given the investment in, the proliferation of and the high level of redundancy of such tools our position is that such a *reflective* piece from an interdisciplinary perspective is overdue in the literature.

The next sections describe examples on the position and application of some selected tools to operate in a wider RG ecosystem (Part 3 and Part 4). Part 5 addresses the issue of risk communication in this domain. Part 6 is a key section as it looks at the potential of data quality assurance systems to improve NM RG. Part 7 examines the continuing role of the Precautionary Principle and how this is captured in tools. Part 8 looks at how *concern* might be incorporated into working tool and this section is followed by concluding remarks.

## Role and status of extant tools in the field

Looking back at the evolution of the development of tools applicable to nanotechnology, the first attempts were focused on control banding (CB) approaches, such as Stoffenmanager Nano. Although ECHA recommends Stoffenmanager, it is necessary to be aware of their uncertainties in chemical safety assessment (Koivisto et al. 2021). It is worthy of note that even-though the evaluated tools were not developed for regulatory use, Stoffenmanager Nano and NanoSafer already include the determinant parameters suggested in ECHA Guidance R.14 and R.14–4, RIPoN-1, RIPoN-2 and thereby principally fulfil REACH requirements for exposure assessment (Ligouri et al. 2016). In general, they were qualitative models whose main goal was to assess and manage the potential risks associated with occupational exposure to NMs. A review by Liguori et al. (2016) of several CB tools, concluded that they were developed for different purposes, used different inputs and the derived risk levels were based on different concepts and assumptions, so a direct comparison of results or an integration in a larger framework was not immediate. At the same time, risk screening tools were developed that have a similar approach, but they also include consumer and environmental RA (Swiss Precautionary matrix, NanoRiskCat (Hansen et al. 2013). In order to enable environmental exposure assessment of NMs in the absence of reliable measurements, several environmental fate models were developed, which can be broadly categorized as “material flow” and “mechanistic” fate models (Meesters et al. 2014; Hristozov et al. 2016).

We have also seen the development of more complex and quantitative RA and management tools, for both environmental and human health RA, leading a to broad offering of RA tools with different degrees of complexity, expertise and guidance requirements. In parallel, other tools with a broader scope than focusing on RA, e.g. LICARA NanoSCAN (van Harmelen et al. 2016), were developed in order to help NMs manufacturers to combine risk and benefit estimations. They may enable and support the implementation of effective risk handling procedures that can be applied despite a lack of full scientific knowledge. However, only some of the methods and frameworks, involve professional end-users, consumers which might be helpful in some situations and unnecessary in others. Later, the need to incorporate economic considerations and consider the whole life cycle of the nano-enabled products, has driven life cycle assessment tools or socio-economic features connected with RA (e.g., SUNDS platform^3^). This included the needs of the insurance industry and their very specific information needs (Murphy et al. 2017). Furthermore, the need to not only assess the risks but provide ways for communicating them, has gained momentum in recent years (Priest 2012; Kühnel et al. 2017; Krug et al. 2018). However, despite the extensive investigation performed in nanosafety and nanotechnologies, the dearth of standardizing methods and tools that satisfy regulatory requirements for scientific robustness and transparency, as well as for establishing benchmarks and guidance for safe use of NMs, has hindered the derivation of appropriate RG models (Trump et al. 2020). There is no doubting the investment in RG models and the considerable expertise that has developed around RG in Nanotechnology. Indeed, Grieger et al. (2019) has proposed, based on nano-risk analysis/governance experiences, efforts to support regulatory decision-making for other emerging technologies are needed as a separate and dedicated research program. Despite the resources invested in the field, there are still several issues to overcome in the area of prediction and characterization of the various effects of NMs. Among the key gaps identified, there is an ongoing lack of consensus in a risk management framework for NMs as well as of certified reference materials and positive/negative controls for NMs, official test guidelines for characterization and toxicity evaluation; methodologies for understanding of the social impacts of NMs, consensus strategies for the transfer of acceptable risk arising from NMs, and finally, proper engagement with stakeholders and society (Murphy et al. 2017; Isigonis et al. 2020).

Rather paradoxically, one of the current problems is the extensive offering of available tools. This creates difficulties for a user outside the nanotechnology research field as many may be redundant in their specific context. Most of these tools were developed within the scope of EU projects and have faced serious challenges in surviving in the world outside the academy and communities of scientific experts. Neither companies, nor regulators trust or understand them totally, making their survival problematic. Besides, the requirement of some kind of regulatory support means there is a persistent reluctance on the part of companies to accept them, (caLIBRAte D2.3).^4^ This is specially complicated as NMs are difficult to regulate due to a lack of information, their complexity, and a regulatory framework tailored for chemicals rather than manufactured materials (Hansen 2017). In fact, the rapid development of nanotechnologies has not been matched by the speed of nano specific adjustments in regulatory frameworks for safety management. This has generated a kind of regulatory gap, whether real or perceived, regarding the proper handling of NMs’ risks (Isigonis et al. 2020). Across emerging technologies in general, this issue has been referred to as a “pacing problem” where regulatory/governance and legal regimes fail to keep pace with the speed of technological development (Marchant et al. 2011). The proliferation and fragmentation of tools has also been considered. This creates a problem around the interconnection between tools. There is a need to establish an interactive system by unifying various tools that allow answers to be given to specific questions. This is what the current nanoinformatics projects are aiming to accomplish, at least for the technical part of the nanosafety assessment (Afantitis et al. 2020; Yu et al. 2021).

The big challenge for establishing a RGF is not only how to select the most appropriate tools among the broad range of existing ones. More challenging still is how to guide the different types of users through the forest of the tools and identifying those that can offer better solutions to their quite different problems. The correct implementation of this guidance should ensure that the risks associated with nanotechnologies, throughout the whole value chain, have been appropriately evaluated and mitigated to an acceptable level. However, existing tools are not all equally helpful in meeting the goals of different stakeholders in a given situation. For instance, commercial actors may seek to have a preliminary assessment available prior to initiating the production of a new nano-based product. The safe by design method offers a step forward in this regard (Jantunen et al. 2021). In this case, as the final product may not yet entirely be designed, large uncertainties in the risk evaluation may be acceptable. For regulators, the lack of enough knowledge in nanotechnologies might result in too high uncertainties about risk and safety. Therefore, ensuring protection may produce unnecessarily prohibitive and costly regulatory measures (Trump et al. 2020). A good example is the recent suggestion for banning all types of carbon nanotubes (Heller et al. 2020). One particular issue that merits some examination in this context is the potential for tools to create a default to the precautionary principle based on data lacunas. Data gaps in the field of NMs are well documented (Marchese Robinson et al. 2016; Trinh et al. 2018; Basei et al. 2019; Furxhi et al. 2020). Their introduction into decision support systems (DSS) tends to short circuit the decision-making process. In effect, the process is stalled in the early data-contingent stage of a continuum. At this stage, precaution becomes the main consideration with a more balanced utilitarian approach held in abeyance until information on NMs increases in granularity (Furxhi et al. 2021) (the operations of the precautionary principle will be discussed in more detail later). All this raises the issue of where tools fit into the overall RG process—which will be addressed in the next section of the paper.

## Where do tools fit in RG?

Any RG process relies on successful crosslinking of resources. Such resources include validated tools and reusable data, information and knowledge, and actors, rules, conventions, processes and mechanisms. All relate to how relevant risk information is converted to sustainable socio-economic benefit and impact (Isigonis et al. 2019; Papadiamantis et al. 2020; Furxhi et al. 2021; Jeliazkova et al. 2021). Moreover, these heterogeneous societal stakeholders may have conflicting interests and indeed perceptions around risk. At their best, tools are neutral resources supportive to the execution of RG tasks. They may on occasion serve as a site of consensus generation (Subramanian et al. 2016).RA frameworks created in the past have been more or less a collection of validated tools and data, linked by descriptive documents or implemented by software driven user interfaces. Tools also have consisted of a web-portal or simply a group of tools and data in publicly accessible repositories (Isigonis et al. 2019; Papadiamantis et al. 2020; Furxhi et al. 2021). The RG ambition with such a bottom-up implementation of RA resources (from data, information and knowledge to decision making) can be expected to be quite limited. With increasing capabilities of AI and machine learning, the question arises, to which extent it is acceptable that such a guidance process implements new boundaries and begins to take over responsibility for decision making with all the opaqueness implicit with algorithmic decision-making and AI (Goodman and Flaxman 2017; Waltl and Vogl 2018; Zerilli et al. 2019; Lindebaum et al. 2020). This may create a lack of trust with such an inverse approach; however, this problem could be mitigated by concepts of explainable AI (xAI), allowing stakeholders to transparently follow the guidance and decision processes.

## Tools and risk communication

According to Renn and Klinke (2015) RG includes “the totality of actors, rules, conventions, processes and mechanisms concerned and how relevant risk information is collected, analysed, communicated and how management decisions are taken.” Communication means analysing key events, processes, and commitments to map the world and make it navigable; communication theory gives us tools to answer empirical, conceptual, or practical communication questions. Thus, communication is a vital symbolic and social process and as such is an integral part of each of Nano-RGF elements.

With regard to RG in general and governance around nanotechnology in particular, how *risk communication* is framed is a contested issue. This has been an important area of debate within the Nanorigo consortium. It is clear that the process of risk communication cannot be viewed as a discreet activity somehow separate from extant power dynamics in a particular field. Moreover, the presence of diverse groups with distinct institutional histories and indeed logics ensures that the practice of risk communication exist within a dense set of social relations. The general position in Nanorigo is that notions of top-down uni-directional communication should be set to one side and that the idea of participation on the part of a wide group of stakeholders in risk communication needs to be privileged. RG is informed by ideas of democracy and inclusion and making this part of any RGF and/or council is an important challenge. The impact of tools on risk communication is such that they can support this instinct for democratisation or these artefacts (which carry their set of values) can lead to a default back to scenarios where experts communicate to passive stakeholders. The presence of tools can lead us to the problem-solving routines of incumbent regimes.

That said, it is clear that tools play an important role in risk communication and indeed this function is an integral part of the RG process related to nanotechnology and nano-related products (Porcari et al. 2019; Isigonis et al. 2020).

Risk communication uses many techniques ranging from conventional media communications, mass community engagement as well as social media. In terms of the risks to scientific endeavour and disinformation this latter element does merit some attention. Murphy et al. (2022) provide an analysis of the representation of NM risk on the Twitter social media platform. Overall working in this area requires a sound understanding of people’s perceptions, concerns and beliefs as well as their knowledge and practices. It also requires the early identification and management of rumours, misinformation and other challenges (Katja Nau et al. 2021). However, we should avoid positing lay groups as passive recipients in any risk communication process. We posit the idea that risk communication is an open two-way exchange of information. This broad definition of risk communication is specified with regard to nanotechnology/nanosafety in an implicit manner, stating that the results of a tool (or model, or approach) should be explained to all users verbally and/or visually (Isigonis et al. 2020). Further, the resulting recommendations for actions should be justified in a comprehensible way, by considering each stakeholder in its specific context. In addition, the magnitude of the associated uncertainty for each result should be clearly communicated (see also precautionary principle in part 7). So overall, a successful message is the aim for communication on NM-related risks. We would add that Miller’s work on explainable artificial intelligence offers a useful set of practices (Miller 2019). At a functional level, the NANORIGO RG platform will include a risk communication platform aiming to provide support to stakeholders. For example, the risk communication platform will link stakeholders to experts that support them in understanding the results obtained from the several tools within a RA and draw appropriate conclusions with regard to risk management.

Most of the tools that are under consideration for inclusion into the RGF already contain some kind of risk communication element to translate the results to the user (*e.g.*, the graphical representation of results used in the NanoApp, https://nanoapp.ecetoc.org/). Further, the output from different tools varies in character and complexity. Hence, the evaluation of results from multiple tools is a challenging task, and the integration of results from different tools into one plausible result will require profound knowledge on the design of each tool and the preceding data collection and evaluation.

The risk communication platform is not developed in isolation but also under consideration of developments in the other RG projects (Gov4Nano, RiskGONE) (Isigonis et al. 2020). The infrastructure project NanoCommons^5^ and the two nanoinformatics projects (NanoSolveIT,^6^ and NanoInformaTIX^7^) work on the integration of different tools, which is expected to result in improved comparability of the output from the tools, or maybe even a uniform output from different tools run in combination. Hence, it will also facilitate the risk communication on nanotechnology and nano-based products in the frame of the RGF. In terms of specific proposals emanating from the Nanorigo consortium around risk communication. We have moved away from seeking to create a Council like entity to an entity we term a “house”. The purpose for this “house” is precisely to provide a forum for open communication between different stakeholder groups. This would exist alongside the *platform* model being developed within the RGF. The “house” model envisaged would seek to mitigate some of the risks of one-way communication and introduce a more Socratic orientation into discussion in the field. The tool sets for risk communication developed by Nanorigo will reflect this orientation.

## A tool to gauge knowledge readiness (KaRL)

Much of the focus on the Nanorigo project around tools has been on an examination of existing tools. That said, within the project we have developed a bespoke too on *data readiness* labelled KaRL. The tool offers an holistic combination of empirical and reflective elements and in this sense may overcome many of the shortcomings of existing tools. The KaRL approach categorizes and guides the assessment of inputs, *i.e.,* resources according to Sen’s capability approach, to outputs needed for RG. The resources needed for RG are the factual knowledge about NM (the scientific input), the individual stakeholders’ needs, the general public concerns (emotions, hopes, fears, apprehensions about the risk) and likely social consequences, economic implications and political responses. The outcomes takes into consideration selection of the type of risk (‘simple’, ‘complexity’, ‘uncertainty’, ‘ambiguity’ according to IRGC 2006, notion on secondary risk) and strategies for approaching risk and proposing a discourse, *i.e.,* instrumental, epistemological, reflective or participative.

The KaRL approach is divided into nine levels. The KaRL 1–3 categories are assigned to a factual dimension of RG (a scientific input). These levels are dominated by natural and technical scientists and use their methods to produce the best estimate of the harm that a risk source may induce, (Jeliazkova et al. 2021).The KaRL 4–6 categories are assigned to the outcome of a co-creation process where experts (knowledge providers) and (individual) stakeholders (problem owners) are involved in a co-creation process. The outcome of the ‘instrumental discourse’ among directly affected groups is functional knowledge to be used by a stakeholder for decision making, communication or participation in RG discursive engagement. The KaRL 7–9 are assigned to the outcome of either a ‘reflective discourse’, which aims to provide a collective reflection about the possibilities for over and under-protection; ‘epistemological discourse’ that aims at finding the best estimates for characterizing the risks under consideration or a ‘participative discourse’ where competing arguments, beliefs and values are openly discussed. The nature of a discourse depends on a type or risk (simple, complex, uncertain and ambiguous). The KaRL7-9 is guided by the ‘common good’ principle. The highest readiness levels could not be achieved without deliberative participatory instruments (*e.g.,* nano-RGC-like structure as a platform for deliberative processes include citizens, consensus conferences, advisory commissions and similar).

KaRL then is both a tool and an approach. It assesses a status and progress of integration of scientific knowledge (scientific and technical discourse) with the diversity of needs and contextual circumstances regarding RG (social discourse) to support risk communication and decision making. When taking analogy with technology *development* readiness levels KaRL approach is about data, information, knowledge, stakeholders and societal needs/concerns *integration* readiness levels for RG.

The urgent need of knowledge (readiness) to address a societal problem have become more visible during the course of NMBP-13 with the advent of the COVID-19 pandemic. According to Beck, the awareness of risk is caused by awareness of the limits of science, rationality, and knowledge. As risk is about the possible consequences of decisions, risk related decisions are of crucial importance. The aim of the KaRL readiness system is therefore to enable participatory science-based decisions about (nano) material risk and reduce the risk of failure in RG. Moreover, the KaRL approach of assessing the status and progress of a decision making process makes the outcome of each step clear and transparent.

## Tools and the spectre of the precautionary principle

The term “precautionary principle” is highly relevant to deal with scientific uncertainty about the full extent of possible harms when extensive scientific knowledge on the matter is lacking. Moreover, the principle is embedded some of the tools we are discussing here. The application is challenging due to the complexity, the assessment of the hazard, research and economic activities. It can be interpreted in many different manners, as there is no generally accepted definition. The literature is quite interdisciplinary and contradictory views exist (Steel 2014). While some argue that the precautionary principle is arbitrary and can be a threat to progress (Cooper 2005), others highlight the possibility to prevent significant threats to the environment and human health (Bronner 2012). According to the European Chemicals Agency (ECHA 2016) there are increasing proofs that the precautionary principle does not limit but supports the development of innovations.

Broadly, in the debate on nanotechnology, we should learn from earlier debates on environmental and societal effects of research. According to John (2007) any use of the precautionary principle should consider practicality and publicity. He outlines that precaution and risk-cost–benefit-analysis are not contradictory but can be considered together. A broader concept which includes technological processes, economic realization, ecological and societal benefits considering the whole life-cycle would be a decisive step forward, and the involvement of all stakeholders would improve the acceptability of precaution. In NANORIGO, several tools were identified addressing the issue of precaution and prevention. However, none of the tool’s deals with all aspects. They cover different aspects and may give different types of information (RA, categorization and grouping), or they may have different modules to evaluate different aspects of risk, as hazard and exposure assessments. There are tools which focus on the regulatory RA approach. Additionally, tools were identified, addressing the precautionary principle in a broader sense. The Swiss precautionary matrix develop by Höck et al. (2018) allows the estimation of 'nano-specific risk potentials' for synthetic NMs and applications for workers, consumers and environment throughout the material's life cycle. It also provides the basis for early decision-making for or against new projects. Besides the chemical assessment, ethical considerations should also be considered in a preventive assessment as already stated in the EC’s Code of Conduct for Responsible Nanoscience and Nanotechnologies Research: “The EC Code of Conduct invites all stakeholders to act responsibly and cooperate with each other, in line with the nanoscience and nanotechnology Strategy and Action Plan of the Commission, in order to ensure that nanoscience and nanotechnology research is undertaken in the Community in a safe, ethical and effective framework, supporting sustainable economic, social and environmental development.”. The diversity in the tools on the one hand and the diversity of the requirements and problems which also change along the life cycle on the other hand, makes it difficult to select the most appropriate tool for the specific requirement. One of main goals of the NANORIGO RG platform will support the different stakeholders in the selection of the appropriate tools to assess the specific NMs/NM-containing products.

The precautionary principle can be a useful pause for thought within an overall governance regime. The problem resides in the potential for a proxy precautionary principle to exist in decision-making tools. Steel (2014) emphasizes the need to step back from the context of more politically weighted criticisms, and instead consider the principle purely in terms of a guideline towards informed decisionality. In any event, this does not offer effective responses to the criticisms laid against the precautionary principle, partly because it is generally held as a means to counter uncertainty.

## Discussion and the integration of “Concern”

The NMBP-13 call topic on RG of nanotechnology that gave rise to the NANORIGO, Gov4Nano and RiskGone projects stated that:

“Significant progress has been achieved in relation to research regarding the safety of engineered NMs and the transfer of this knowledge into regulation. Still, more needs to be done as nanotechnology reaches the market. To fill this gap, transdisciplinary RG is required based on a clear understanding of risk, its management practices and the societal risk perception by all stakeholders.”

The call text expresses the reliance of RG not just on RA and management, but importantly, on *“the societal risk perception by all stakeholders”*. But what exactly does that imply? What does it mean to base RG on the societal risk perception of all stakeholders—and how can it be realized? More specifically, what are the role of decision-making tools in such a scenario.

A review of existing governance frameworks and tools suggests that despite significant funding for the development of RA methods and tools in the EC’s NMBP program over the last 15 years or so, only a few projects have tackled the question of integrating societal perspectives.^8^ The most notable examples are LICARA, SUNDS and caLIBRAte, but these projects focus mostly on ‘narrow’, quantitative societal indicators like economic or health benefits or breakthrough potential and do not offer clear roadmaps for integrating stakeholder perceptions. In contrast, the *Science with and for Society (SwafS) program*^9^ has funded a range of Coordination and Support Actions on stakeholder engagement in the context of Responsible Research and Innovation, but these mostly describe broad-based approaches that have only an indirect bearing on the world of nanotechnology governance.^10^ Notably, there are existing regulatory approaches like the socio-economic analyses performed by ECHA’s scientific committee, but these approaches have been criticized for focusing primarily on economic rather than social analysis.^11^

In short, the question on how to integrate societal perspectives in RG and indeed in RG tools still deserves attention. There remains a ‘societal gap’ in RG. The three NMBP-13 projects offer an excellent opportunity to bridge that gap. Indeed, the NANORIGO Nanotechnology RGF takes an inclusive perspective, basing the governance process on three types of knowledge: Scientific technical evidence; Evidence about the perception and concerns, expressed by various actors; stakeholders; and Knowledge about the context and culture, such as the regulatory culture or regional preferences, in which the technology-based products are developed.^12^ Within NANORIGO, there has been focused work taking place under the umbrella of *concern* – forming part of the RGF. It is certainly interesting to consider how the idea of *concern* might be captured in a set of NM governance tools. Overall, this set of deliverables underlines that RG provides a process: a comprehensive and harmonized guidance for early identification and handling of risks, involving multiple stakeholders. This approach chimes with the International RG Council by Ortwin Renn (2006). IRGC White Paper on RG: Towards and Integrative Approach. The authors make the following argument;

“Risk appraisal thus comprises a scientific assessment of both the risk and of questions that stakeholders may have concerning its social and economic implications. [...] Equally important to understanding the physical attributes of the risk is detailed knowledge of stakeholders’ concerns and questions – emotions, hopes, fears, apprehensions – about the risk as well as likely social consequences, economic implications and political responses”

It is interesting to consider the relationship between *emotional* conditions such as concern or even fear and so-called tools. Tools may offer reassurance in some cases. That said, there is a danger that tools may also obscure human subjectivities and instead offer a somewhat illusory objectivity.

Clearly, it is no small matter to effectively integrate these different types of knowledge in a single, coherent framework. This is in part due to the incommensurability of ‘hard’ scientific data with ‘soft’ qualitative data. Due to their complex, contested and inherently qualitative nature, broader societal perspectives do not lend themselves to integration in decision support tools which require accessible, quantifiable, reproducible and more or less uncontested indicators as input. RG is more than RA: decisions to promote, inhibit or otherwise regulate new nano-enabled products or processes are not fully determined by the quantitatively identifiable risks, but are shaped by a host of wider economic, environmental and socio-ethical considerations. Scientific evidence is but one piece of the governance puzzle. If it is to engender trust among citizens and stakeholders, the RGF should address these wider concerns alongside risk. So, the question: *“What is up for discussion?”* is important. If the RGF and related toolsets only considers risks per se, then other, legitimate societal concerns about the governance of innovation are not up for discussion. Yet the integration of scientific insights with social evidence is exactly what is required to effectively govern nanotechnologies.

The challenge is to create tools that can underwrite ‘socially robust’ governance framework that includes a capacity to anticipate and respond to broader ethical and societal concerns. Such a model could also define a ‘niche’ for the future RGC in the landscape of existing institutions. One of the challenges for groups of researchers in the field of NRG is how do tools fit into this ambition. The potential benefits of integrating societal perspective have been demonstrated time and again: it enhances stakeholder trust and gears innovation towards sustainability rather than performance. It is the remedy for the trust deficit that plagues research and innovation policies. But for all its expected benefits, there is still a dearth of effective approaches for integrating the outcomes of concern assessment in policy decisions. Insights gained during these events have not translated in effective mechanisms for integrating societal perspectives (apart from the establishment of the nanotechnology observatory which mostly monitors technological developments). In an adjacent domain, Čartolovni et al (2022) have examined the difficulties around integrating ethical, legal and social considerations into data driven decision support tools. Their extensive literature review in the area of decision-making tool in medicine point to a number of problems areas that are common in multiple fields. These include bias, trustworthiness and opacity. Such potential shortcoming would also have to be addressed in those tools utilised in any RG process in the area of nanotechnology.

## Closing Remarks

Assuring safe and sustainable nanotechnology is a complex multi-agent process, which requires the integration and balancing of several interdisciplinary aspects. Following such a path is indispensable if we desire the innovations in the nano-field to evolve from emerging technologies to applications and products that provide benefits while minimizing the risks for society and the environment. While the private sector is one of the main drivers in bringing innovations to the market, the subsequent steps for the integration of such innovations in society involve multiple actors, which contribute to the discourse with diverse inputs and needs. This translates not only into the production of information, but also in the development of tools that, based on such knowledge, can aid other stakeholders in integrating scientific information in their decision-making process. One consistent problem in the field has been the issue of incomplete and inconsistent information, the KaRL approach outlined above will assist in this regard. Overall, to achieve a responsive and democratic set of practices for the governance of nanotechnology related risk, a reflexive disposition towards such artefacts will be required – one that lends a greater understanding of the risk posed by decision making tools as well as their benefits. Insights from the management and social science disciplines alert us to the presence of pre-existing logics, the danger of reification and embedded ethical positions in such tools. This is a complex area with tools having multiple functions from processing and categorising data to risk communication.

Different levels of uncertainty caused by limited scientific knowledge call for specific methods and tools, for example by using probabilistic models or relative ranking systems (Som et al. 2013). In the European Union, such approaches are guided by the precautionary principle, which, from a political point of view, defines the risk acceptability in the absence of complete information (De Marchi 2003). Adaptability of any system is needed also to integrate and fully make use of future information/tools/data, for example the advent of big data and AI. This element will require further elucidation by scholars working in the area of RG around emerging technologies due to the danger associated with the so called “black box”—namely opaqueness and reification (Linkov et al. 2018). Decision-making tools have a role to play in any new governance regime but there remain doubts around their effectiveness. The vast majority of tools are never used and fall into obscurity. Moreover, tools are scattered across fields of interest (toxicology, environmental assessment, LCA, etc.) and are very difficult to combine into a single approach that prospective users can actually work with to guide their decisions. Overall, despite the plethora of tools for RA there is a dearth of ‘tools’ (or approaches) to integrate societal considerations, this governance component is underdeveloped in current RG models.

## Footnotes

1 https://www.gov4nano.eu/wp-content/uploads/2019/08/NMBP-13-poster-ENF2019-v2.pdf (Accessed November 2021).

2 Check for reference here.

3 https://sunds.gd/ (Accessed November 2021).

4 See Comin, D. and Giarda, M., 2019. D2. 3 Scientific paper on final results of the model specification, parametrisation, and calibration of multi-sector extension of baseline model. https://ftp.zew.de/pub/zew-docs/veranstaltungen/FRAME/Deliverables/D2.3.pdf (Accessed November 2021).

5 https://www.nanocommons.eu/ (Accessed November 2021).

6 https://nanosolveit.eu/ (Accessed November 2021).

7 https://www.nanoinformatix.eu/ (Accessed November 2021).

8 *See: NANORIGO D1.8—Critical evaluation of governance frameworks—considering the current integration of ethical, environmental, social, economic, legal and/or regulatory considerations. DPF, 28 August 2020. *https://nanorigo.eu/wp-content/uploads/2020/08/NanoSafety-Cluster-NEWSLETTER-21-December-2020.pdf*.*

9 https://ec.europa.eu/programmes/horizon2020/en/h2020-section/science-and-society (Accessed November 2021).

10 *Already in 2007 and 2008, the Nanotechnology Safety for Success dialogues organised by the European Commission addressed the question on how to organise a stakeholder dialogue around nanotechnology (*https://ec.europa.eu/health/dialogue_collaboration/events/ev_20081002_nl*), but the insights gained during these events have not translated in effective mechanisms for integrating societal perspectives (apart from the establishment of the nanotechnology observatory which mostly monitors technological developments).*

11 *See: Lost at SEA—The information policymakers actually need from applicants and SEAC opinions. ChemSec, 2019: *https://chemsec.org/app/uploads/2019/02/Lost-at-SEA-ChemSec-2019.pdf*.*

12 *See: NANORIGO Milestone 34—OUTLINE of the NANORIGO Nanotechnology Risk Governance Framework (NRGF). EPFL, 19 December 2019.*
